# Effects of microwave time on quality of grass carp fillets processed through microwave combined with hot‐air drying

**DOI:** 10.1002/fsn3.1708

**Published:** 2020-07-15

**Authors:** Jiaying Qin, Zhihe Wang, Xichang Wang, Wenzheng Shi

**Affiliations:** ^1^ College of Food Science and Technology National R&D Branch Center for Freshwater Aquatic Products Processing Technology (Shanghai) Shanghai Ocean University Shanghai China

**Keywords:** grass carp, microwave combined with hot‐air drying, microwave time, quality, taste

## Abstract

In this study, the quality changes of grass carp fillets dried by microwave‐hot‐air combined drying under different microwave time were investigated. The salted fillets were dried at 385 W microwave with different time (0, 2, 4, 6, 8, and 10 min), followed by 65℃ hot air to the end. The quality of fillets was evaluated by drying time, color, hardness, rehydration ratio, and taste characteristics (ATP‐related compounds, free amino acids, E‐tongue taste profile, EUC, and TAV). Results showed that grass carp fillets dried by microwave‐hot‐air combined drying had better qualities compared with single hot‐air drying. Besides, microwave time had obvious effects on the quality changes of grass carp fillets, and 6 min was considered as the optimal drying time.

## Graphical abstract

In this study, the quality changes of different microwave time (0, 2, 4, 6, 8, and 10 min) combined with hot‐air drying grass carp fillets were investigated. The quality of fillets was evaluated by drying time, color, hardness, rehydration ratio, and taste characteristics (ATP‐related compounds, free amino acids, E‐tongue taste profile, EUC, and TAV). Results showed that microwave combined with hot‐air drying grass carp fillets had better qualities compared with single hot‐air drying, and 6‐min microwave drying time was considered the optimal drying time.

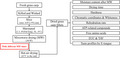

## 1 INTRODUCTION

Fish and fish products are important sources of high‐quality protein and other nutrition such as Omega‐3 fatty acids, vitamins, and minerals, of which the production and consumption increase sharply over the years. Grass carp (*Ctenopharyngodon idellus*) is one of the most important commercial freshwater‐cultured fish species in China. The production of grass carp always ranks first among freshwater farmed fishes in China (China Fisheries Report, 2013–2018). It is popular in China for its short growth cycle, easy feeding, low price, high nutritional value (Wang, Zhu, Zhang, Wang, & Shi, 2018) and palatability (Akondi et al., 2014). However, the high water activity, abundant nutrients, and neutral pH in the fish muscle make it easy to deteriorate, oxidize and rot in a short time (Sampels, 2015), resulting in great commercial loss. Drying is one of the important methods to prolong the preservation time applied in grass carp by reducing Aw to inhibit the enzyme activities and limit the growth of microorganisms. Furthermore, compared with refrigerated storage, drying processes form special flavor, helping improve taste, sensory, digestibility, and safety characteristics of grass carp (Siddhnath et al., 2020).

At present, traditional natural drying and hot‐air drying are mainly used for dried grass carp. Besides, many new drying methods have also been developed and used in aquatic products, such as microwave drying (Fu, Lin, Xu, & Wang, 2015), vacuum freezing drying(Ma, Qu, & Sun, 2017), hot pump drying (Li et al., 2017), and infrared drying(Deng et al., 2014). However, nearly every drying method has its disadvantage. For example, natural drying is greatly affected by external influences, which makes production efficiency low. The poor sanitary conditions make it difficult to adapt to the growing needs of food quality and safety systems (Bala & Mondol, 2001). The insufficiency of hot‐air drying is the long‐time and high‐energy consumption, browning effect, possible nutrition loss, and quality deterioration in color and texture (Izli, Taşkın, & İzli, 2019). Microwave drying uses high‐frequency electromagnetic waves as a heating source and microwave energy directly converts into thermal energy inside the material (Al‐Harahsheh, Al‐Muhtaseb, & Magee, 2009). Compared with other dry methods, microwave drying is a highly efficient internal heating form, which greatly shortens the drying time and saves more energy. Whereas, the inhomogeneous heat distribution and lack of obvious surface changes in materials make it difficult to decide the drying terminal point. Besides, products may shrink after microwave treatment (Wang, Tan, Han, & Gu, 2012).

To overcome these limitations, combined drying methods are applied. Combined drying refers to a composite drying technology that uses two or more drying methods to complement each other according to the characteristics of the materials. Hot‐air drying is prone to case hardening, which is not conducive to the drainage of internal moisture, while microwave drying could effectively promote the drainage of material moisture. By combining the hot air and microwave drying technologies, the drying time and efficiency will come to a balance. Mohd and Ng (2011) found that dried catfish slice with a microwave‐hot‐air dehydration system took less drying time and energy consumption compared to drying with hot air convective alone.

Quality of the dried products is determined by changes in color and texture from the fresh products as they are closely allied with consumer preference (Seerangurayar, Abdulrahim, Al‐Ismaili, & Jeewantha, & Nasser, 2019). Microwave combined with hot‐air drying can also effectively improve the color and texture of dried aquatic products. Meng, Liu, Wu, and Li (2015) found that Microwave‐hot‐air combined drying had obvious advantages for oyster drying, compared with hot‐air drying, microwave drying, and vacuum drying. The combined dried oyster has better performance in color, elasticity, shrinkage, rehydration, physical, chemical, and sensory properties. Weng, Jiang, Liu, and Wang (2017) optimized the red shrimp microwave‐hot air combined drying process and found that the combined drying was significantly better than single microwave and hot‐air drying in terms of the chroma of the dried product and the hardness, elasticity, and chewiness after rehydration.

Despite more and more microwave‐hot‐air combined drying was studied, most of them still focus on the plant foods (Yao Tan et al., 2019). There were few reports on grass carp which is considered as the most thriving aquatic product in China. The effects of single microwave drying and hot‐air drying on drying characteristics, drying kinetics (Qi, Peng, Cheng, & Jin, 2016), nutrition, and flavor characteristics (Wu & Mao, 2008) of dried grass carp were systematically studied, providing a theoretical basis for further exploration of microwave‐hot‐air combined drying. Different microwave time could significantly affect the quality of dry products (Yao et al., 2019). Long‐time microwave drying would cause products scorch, but short‐time microwave drying cannot effectively improve drying efficiency. Therefore, this study optimized microwave time in combined drying to get dried grass carp fillets with better quality and provide theoretical guidance for industrial production.

## 2 MATERIALS AND METHODS

### 2.1 Samples preparation

Twelve cultured grass carps (weight 2.9 ± 0.3 kg, length 61.3 ± 1.5 cm) were purchased from a nearby supermarket（Shanghai, China）in November 2019 and then transported to the laboratory alive within 30 min. The carps were killed, decapitated, descaled, and gutted. The spines of carps were removed, and the remaining parts were evenly cut in half, and further cut into 8.0 cm × 2.5 cm × 1.0 cm fillets. Food grade iodine‐free salt crystals were dissolved in pure water at 20°C to obtain 8% (by mass) salt solution. The fish fillets were immersed in the brining solution (1:3 fish to solution by mass) without stirring for 3 hr at 4 ± 1°C.

Every 200 g of salted fillets were drained and laid in MM721NG1‐P1150 microwave oven (Midea) for microwave drying with 385 W power for different time (2, 4, 6, 8, 10 min) according to the results of preliminary experiments. Then, they were immediately placed in a digital constant temperature hot‐air drying oven (Demashi) and dried by 65℃ hot air. In addition, conventional hot‐air drying was performed as control group (0‐min microwave time) with the same experimental conditions.

### 2.2 Drying time

The fillets with different microwave drying time were then dried by hot air. During this process, the moisture content of the fillets was measured every half an hour. The drying process was not stopped until the moisture content dropped to 30.0 ± 1.0%. The total drying time included microwave drying time and hot‐air drying time.

### 2.3 Moisture content

Moisture content determination of fillets samples referred to the current GB 5009.3–2016. The petri dishes were constant weight before the experiment. The fish fillets were crushed evenly, then accurately weighed about 2.00 g by EL204 analytical balance (Mettler Toledo), and tiled evenly in a petri dish. With the lids tilted aside, the petri dishes with the sample were placed in a blast dryer and dried at 105°C to constant weight. Three repeats for each sample were taken. The value of moisture content is calculated using the following formula:

$$X=\frac{m_{2}-\left(m_{3}-m_{1}\right)}{m_{2}}\times 100$$

where *X* stands for the moisture content in the sample of the mixture in terms of %; *m*_1_ is the mass (g) of the petri dish; *m*_2_ is the mass (g) of the sample; *m*_3_ is the mass (g) of the petri dish and sample after constant weight drying.

### 2.4 Hardness determination

The hardness of fillets was determined according to Yao's methods (Yao et al., 2019) with some modifications to fit the samples. Hardness determination was performed on the dry fillets by TA‐XT Plus (Stable Micro System, U.K.). The determination was performed by a 2 mm diameter flat‐head stainless steel cylindrical probe (P/2) with the distance at 3.000 mm compression. The speeds of pretest, test, and post‐test were 0.50, 0.50, and 10 mm/s, respectively. Eight fillets were taken as a measurement sample in each group, and the detection was performed three times at random at three different positions of each fillet.

### 2.5 Color determination

The color of fillets was determined by a portable colorimeter CR‐20 (Konica Minolta through three chromatic coordinates (L*, a*, and b*) of the CIELAB color space system. The standard white reflector was used to calibrate colorimeter before determination. L* represents brightness; the larger the value, the brighter the sample. a* represents the red‐green degree; the positive value represents red, and the negative represents green. b* value represents the yellow‐blue degree; the positive value represents yellow, and the negative represents blue. Whiteness (W) is calculated from the L*, a*, and b* as below:

$$W=100-{\left[{\left(100-L^{*}\right)}^{2}+a^{*2}+b^{*2}\right]}^{1/2}$$

where L*, a*, and b* were determined from sample. The higher the whiteness, the whiter the sample (Ma, Shi, & Wang, 2016). In order to reduce the uneven color of grass carp fillets, 8 fillets were taken as a measurement sample in each group, and the detection was performed three times at random at three different positions of each fillet.

### 2.6 Rehydration ratio

The rehydration ratio was determined according to the previous procedure (Monteiro, Link, Tribuzi, Carciofi, & Laurindo, 2018) with slight modifications. Dried fillets weighed with electronic balance were put into pure water in a water bath to rehydrate for 30 min at 90°C, then drained on a single‐layer stainless steel wire mesh for 15 min and weighed again. Four replicates were performed for each sample. The rehydration ratio is calculated using the following formula:

$$R_{\text{reh}}=\frac{m_{r}}{m_{t}}$$

where *R*_reh_ is the rehydration ratio of sample; *m_r_* stands for the weight of sample after rehydration; *m_t_* stands for the weight of sample before rehydration.

### 2.7 ATP‐related compounds

ATP‐related compounds were extracted from samples according to previous methods with slight modifications (Yang, Shi, Zhou, Qu, & Wang, 2019). Each sample was weighed 5 g and homogenized for 30 s with 15 ml cold perchloric acid (10% by volume) by a FM‐200 homogenizer (Shanghai Fokker Equipment Co. Ltd.) and then centrifuged via a H2050R high‐speed freezing centrifuge (Changsha Xiangyi Co. Ltd.) at 10,000 × g for 15 min. The sediment was washed thoroughly, then shocked evenly with 5 ml cold perchloric acid (5% by volume) and centrifuged in the same condition. This process was repeated twice. The supernatants were mixed, adjusted to pH 6.5 (with 1 M and 10 M KOH), left to stand for 30 min, diluted to 50 ml (with high‐purity water), and filtered through 0.45 μm aqueous phase membranes. All the above‐mentioned operations were conducted at temperatures below 4℃. The filtrates were analyzed by W2690‐2998 high‐performance liquid chromatography (Waters Co.), and COSMOSIL 5C18‐PAQ liquid chromatography column and SPD‐10A (V) detector were equipped. Phosphate‐buffered solution (0.05 M) with pH 6.5 and methanol were used for equal elution for 20 min at a volume ratio of 92:8 at a flow rate of 1 ml/min. The injection volume was set to 10 μl, and the wavelength detected was 254 nm.

### 2.8 Free amino acids (FAAs)

The method from Wang (Wang et al., 2012) was used for pretreatment of FAAs. 2 g sample was fully homogenized with 15 ml trichloroacetic acid solution (15% by mass), and left for 2 hr, then centrifuged at 10,000 × g for 15 min and filtrated. 5 ml of supernatant was taken to adjust pH to 2.0 (with 3 M NaOH), then diluted to 50 ml (with high‐purity water), and filtered through 0.22 μm aqueous phase membranes. All operations were conducted below 4°C. Determination and analysis of FAAs were performed by L‐8800 automatic amino acid analyzer (Hitachi). All analyses were done in triplicate. The identification and quantification were conducted using the retention times and peak area ratios of each FAA standard and the sample, respectively (each of 17 FAA standards in one solution).

### 2.9 Taste‐active value (TAV) and equivalent umami concentration (EUC)

TAV was calculated as the ratio between the concentration of one taste compound and its threshold. The taste thresholds were referenced from the literature (Chen & Zhang, 2007). If TAV ＞ 1, the compound could independently contribute to the overall taste profile. Furthermore, the higher the TAV, the greater the contribution. TAV is a relatively objective evaluation method widely used in various flavor studies, but it cannot comprehensively evaluate the interaction between various taste substances, such as synergistic effect and offsetting effect (Scharbert & Hofmann, 2005).

Yamaguchi performed sensory experiments on the interaction between taste 5'‐nucleotides and FAAs and proposed a monosodium glutamate equivalent evaluation model called EUC, which was used to indicate the synergistic effect between them (Yamaguchi, Yoshikawa, Ikeda, & Ninomiya, 2010). The 5'‐nucleotides that play a role in enhancing umami are IMP and AMP in grass carp, and the UAAs are Asp and Glu (Chanvorleak, Moon, & Lee, 2016). EUC is calculated by the following formula (Yamaguchi, Yoshikawa, Ikeda, & Ninomiya, 1971):

$$\text{EUC}=\Sigma a_{i}b_{i}+1218\left(\Sigma a_{i}b_{i}\right)\left(\Sigma a_{j}b_{j}\right)$$

where EUC is in terms of gMSG/100 g; *a_i_* is the concentration (g/100 g) of each umami amino acid in fillets, Asp or Glu, and *b_i_* is the relative umami equivalent concentration (RUC) for each umami amino acid compared to MSG (Glu, 1; Asp, 0.077); *a_j_* is the concentration (g/100 g) of each umami 5′‐nucleotide in fillets, IMP or AMP, and *b_j_* is the RUC for each umami 5′‐nucleotide compared to IMP (IMP, 1; AMP, 0.18); 1,218 is a synergistic constant.

### 2.10 Electronic tongue (E‐tongue)

The E‐tongue (Alpha M.O.S. of ASTREE, France), composed of a taste sensor array, a signal acquisition system and a pattern recognition system, was normally used to profile the taste of grass carp (Khaydukova, Cetó, Kirsanov, Del Valle, & Legin, 2015; Yuhei, Junpei, Rui, Hidekazu, & Kiyoshi, 2016). Before testing, the sensors of E‐tongue must be successfully adjusted and calibrated by 0.01 M HCl. The fillets were weighed 2.00 g, homogenized with 25 ml deionized water, and then allowed to stand for 30 min. After centrifugated at 10,000 × *g* for 10 min below 4°C, the filtrate was diluted to 100 ml with high‐purity water for further tested. There were 5 reference solutions tested by E‐tongue to help characterize the taste profile of each group. They were respectively 6 mM MSG, 6 mM NaCl, 5 mM citric acid, 20 mM sucrose, and 6 mM quinine, relatively representing umami, salty, sour, sweet, and bitter (Dang, Gao, Ma, & Wu, 2015). The 80 ml sample solution, consisting of 5 ml test solutions and deionized water, was poured into a dedicated sample cup and then tested by E‐tongue at 20℃. Each sample was analyzed seven times, and the last three times were used as raw data for principal component analysis (PCA). During the measurement, the data collection time for each sample was 120 s, and the collection frequency was 1 s each time. The response value on each sensor during the collection time is used as raw data of the electronic tongue. Clean the sensor with deionized water after each measurement for data reliability.

### 2.11 Statistical analysis

The variance of the data expressed as mean values ± standard deviations. One‐way analysis of variance (ANOVA) with LSD method was carried out using SPSS 22.0 (SPSS Inc.), and significant difference was expressed at *p* < .05 level.

## 3 RESULTS AND DISCUSSION

### 3.1 Moisture content of fillets after microwave and Drying time

**FIGURE 1:**
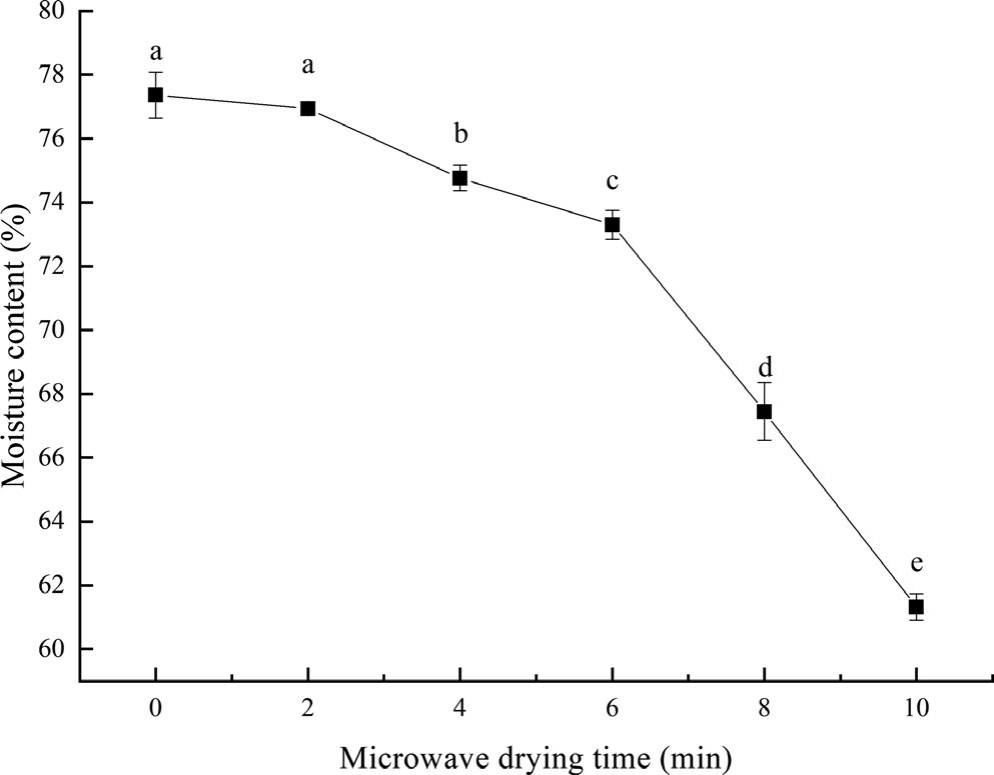
Effects of microwave drying time on moisture content of grass carp fillets after microwave drying. The same index letters indicate that the mean values are not significantly different at a confidence level of 95% (*p* < .05)

Moisture contents of grass carp fillets treated with different microwave drying durations were shown in Figure 1 decreased along with microwave drying time, and the fillets without microwave drying treatment (0 min) had the highest moisture content of 77.36%. Similar results were stated by Zhang et al. on microwave‐drying tilapia fillets and Osman et al. on rainbow trout fillets. According to the principle of microwave drying, water molecules change directions quickly after absorbing microwave energy, then generate intense frictions and violent collisions between molecules, and release a large amount of heat. The total microwave energy absorbed increased along with microwave drying time, promoting the rapid evaporation of moisture. Besides, the fillet itself absorbed microwave energy, which also helped moisture evaporate and shortened the total drying time (Maskan, 2006).

**FIGURE 2:**
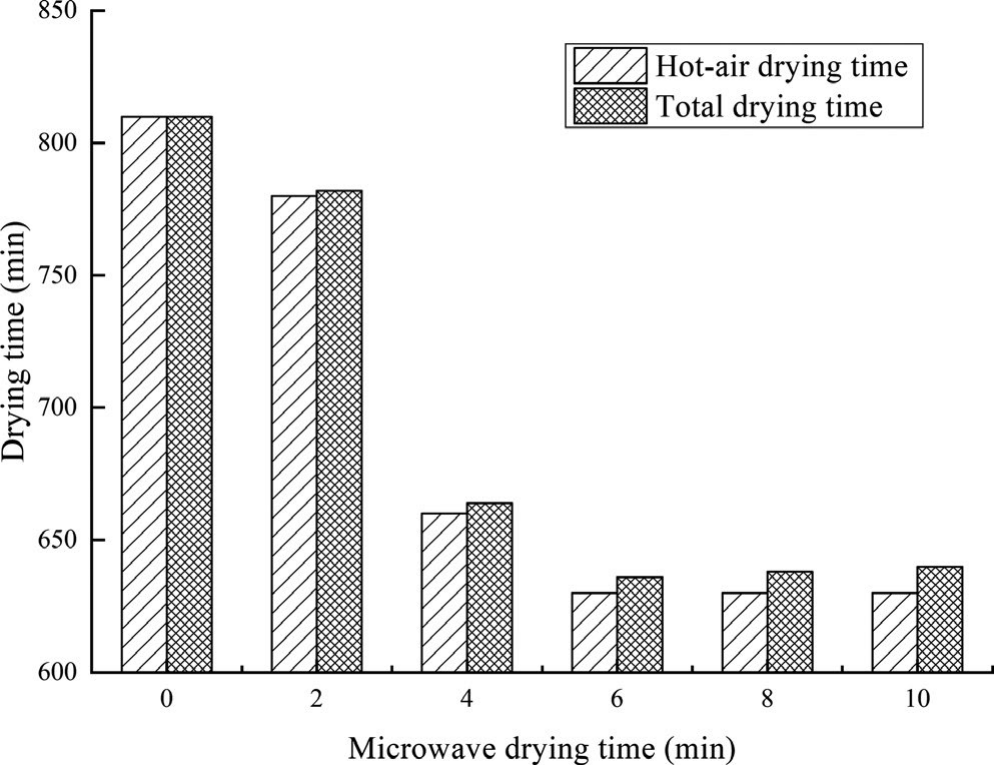
Effects of microwave drying time on hot‐air drying time and total drying time of dried fillets

The total drying time changed with microwave drying time in combination drying and was displayed in Figure 2, which was used to evaluate the efficiency in time and energy of the drying methods. The single hot‐air drying (0 min) took the longest time of 810 min. It indicated that microwave‐hot air combined drying, compared with single hot‐air drying, was more efficient and time‐saving. Qi's study (Qi et al., 2016) also indicated that grass carp could be dried much faster by microwave drying (200 ~ 400 W) at the same time, compared with hot air (60 ~ 70°C). When microwave drying time was less than 6 min, the total drying time reduced sharply with the increase of microwave time. But then total drying time did not decrease and even slightly rose with increasing microwave time, because of the same hot‐air drying time and different microwave drying time. The temperature rose very fast due to long microwave drying time, causing the unbalanced moisture distribution. The water on surface evaporated too rapidly, while the internal transported relatively slowly. The surface muscles were overheated, shrank, and harden, preventing internal moisture from evaporating. Thus, the 6‐min microwave‐drying fillets had the least total drying time (636 min).

### 3.2 Hardness of dried fish fillets

**FIGURE 3:**
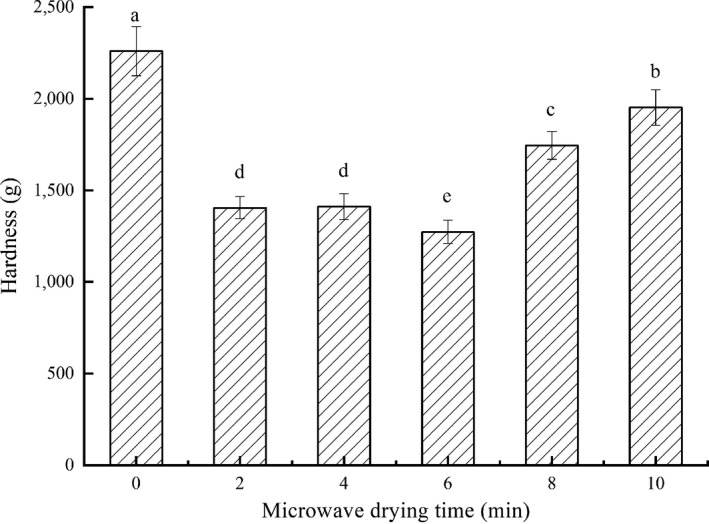
Effects of microwave drying time on hardness of dried fillets. The same index letters indicate that the mean values are not significantly different at a confidence level of 95% (*p* < .05)

The effects of microwave drying time on hardness of fillets were shown in Figure 3. The drying process would greatly make the fish's muscles hardened and particularly tough, unacceptable to consumers (Seerangurayar et al., 2019). In this study, the hardness of each group was more than 1,200 g, much harder than the fresh. With microwave drying time increasing, the hardness showed the two trends: first decreased and then increased. The fillets with 6‐min microwave drying were the softest, but those with 0‐min were the hardest. Hence, combined drying could reduce the excessive hardness compared with single hot‐air drying.

The connective tissue composed of elastic fibers and collagen fibers in fish meat was the main factor to determine the hardness (Chen, Hu, Bao, Zhou, & Li, 2019). The higher the fibers content and the greater the cross‐linking degree, the harder the fillets. Both the increasing temperature and the extending drying time aggravated protein degeneration and shrinkage, causing the increasing degree of cross‐linking in fillets’ muscles, and leading to the increasing hardness (José, Amparo, & Pedro, 2003). Moreover, the hardness of dried fish was proved to be positively correlated with the drying rate by single drying methods in previous study (Chen et al., 2019). During the drying process, when the rate of the internal moisture diffused to the surface was lower than the rate of the surface moisture to evaporate, the fillets were easy to wrinkle and form a dense hard shell.

### 3.3 Color of dried fish fillets

**FIGURE 4:**
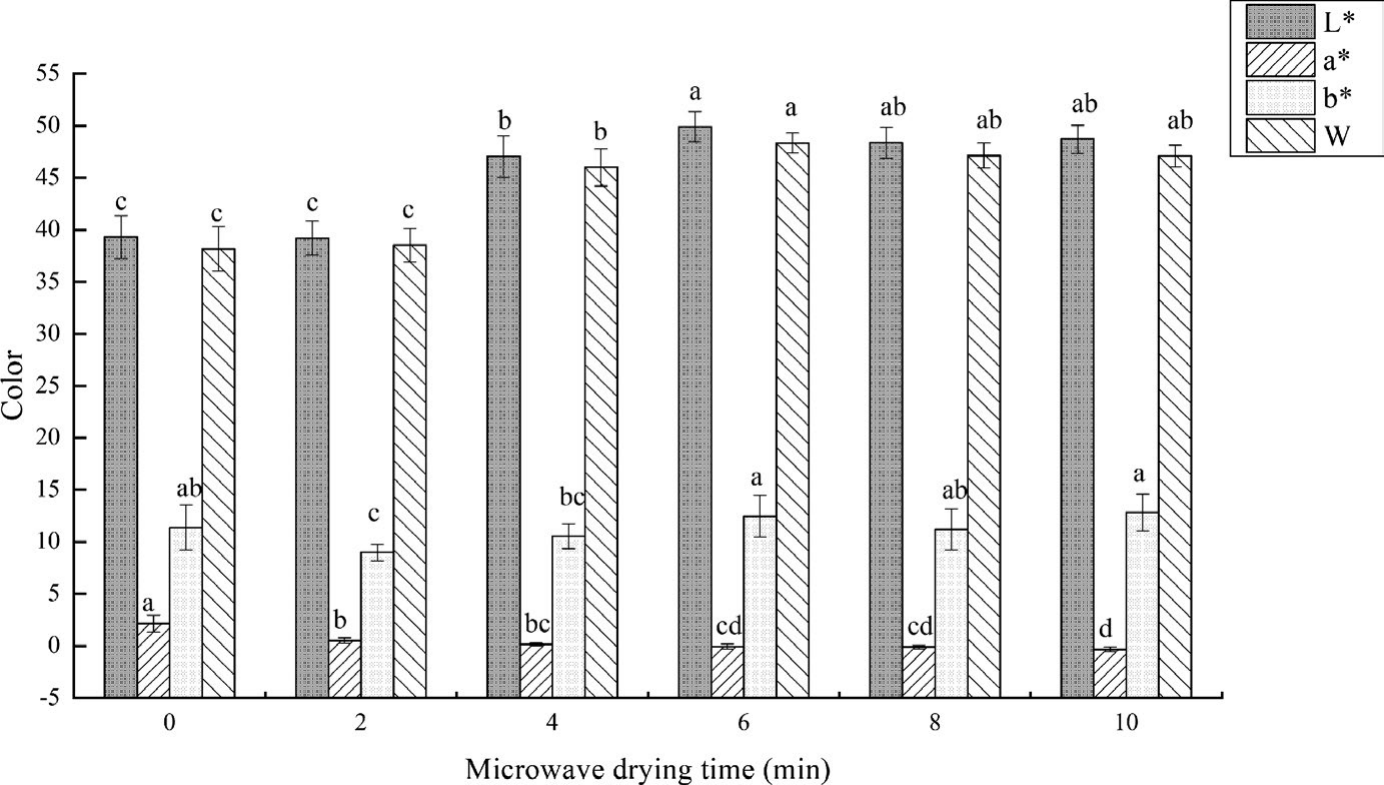
Effects of microwave drying time on chromatic coordinates L*, a*, b*, and W (whiteness) of dried fillets. The same index letters indicate that the mean values are not significantly different at a confidence level of 95% (*p* < .05)

The color changes of fillets under different microwave drying time was shown in Figure 4. The L^*^ and W changed simultaneously, first increased and then decreased with the extension of the microwave heating time. They reached the highest values in 6 min microwave‐drying fillets, but the lowest in 0 min and 2 min fillets. Appropriate microwave drying combined with hot‐air drying effectively made the fillets whiter and brighter. The same brightness enhancements were observed in microwave‐assisted hot‐air dehydration treatment catfish slices by Mohd et al.

Changes on color in fillets were caused by thermal denaturation of proteins. The abundant proteins and amino acids in fillets were heat‐treated, and their multi‐level structures were forced to change (Guizani, Obaid Al‐Shoukri, Mothershaw, & Rahman, 2008). Appropriate thermal denaturation made fish muscles mature, resulting in the light scattering characteristics of the surface, and creating white and gloss that was preferred by consumers, which manifested in the increase of L* and W. Conversely, excessive thermal denaturation easily caused the aging of fillets, manifested by rapid dehydration, severe contraction, and deformation of muscle fibrin, which made the fillets dark and dull, sometimes resulting in severe burnt. Moreover, the Maillard nonenzymatic browning reactions also explained the changes on color (Rahman, 2006), which easily occurred under high‐temperature and long‐time conditions with rich amino compounds and carbonyl compounds in fillets. The changes in L* of dried samples were used as a measure of the degree of browning (Ávila & Silva, 1999). Combined with Figure 2, the increase in total drying time provided the long‐time condition, and the increase in microwave time provided the high‐temperature condition, which were consisted to the decrease of the L*. The a^*^ decreased with the increase of microwave drying time and the b* did not show obvious regularity in this study. From the degree of data change, the changes of L* and W were more obvious compared with a* and b*.

### 3.4 Rehydration rate of dried fish fillets

**FIGURE 5:**
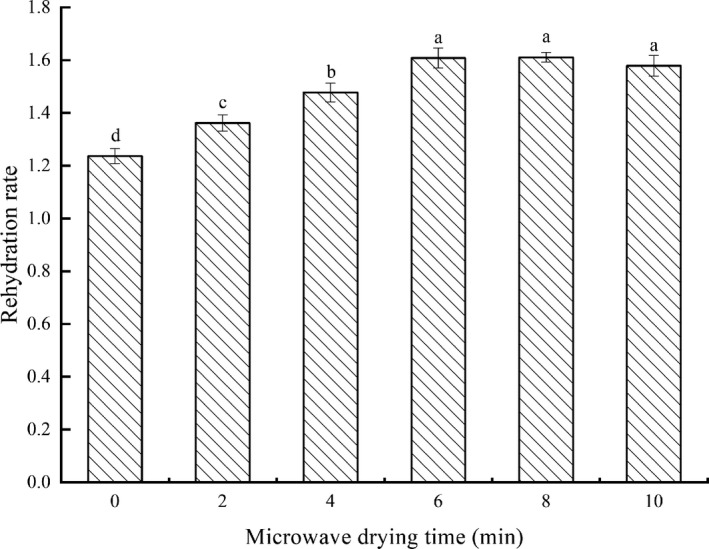
Effects of microwave drying time on rehydration rate of dried fillets. The same index letters indicate that the mean values are not significantly different at a confidence level of 95% (*p* < .05)

During the drying process, if the cells in the material are irreversibly damaged, causing the cells to lose their integrity and the tissue structure to collapse, the rehydration rate of the product will reduce, indicating that the quality of the dried product is poor. Therefore, the rehydration rate is always considered as an important indicator in evaluating the quality of dry products (Hamza, Ahsen, Emine, Gülhan, & Taner, 2019). Figure 5 showed the effects on the rehydration rate of grass carp fillets dried by different microwave drying time. The rehydration rate of 0 min microwave‐drying fillets was the lowest, and those of 6, 8, and 10 min showed no significant difference (*p* > .05) and were the highest. The rehydration rates of combined drying were all higher than that of single hot‐air drying. It was illustrated that microwave combined with hot‐air drying could reduce the irreversible structural damage. Further, the rehydration rate increased along with microwave drying time. When the microwave drying time was 6 min or longer, there was no significant difference (*p* > .05) in the amount of irreversible structural damage in these fillets. The rehydration rate was considered to be closely related to the drying method (Chen et al., 2019). In Medeni's study on drying kiwifruits, the hot‐air combined microwave drying method exhibited better rehydration characteristics than single drying method (Medeni, 2001), which was consistent with the result in this study.

### 3.5 ATP‐related compounds

**FIGURE 6:**
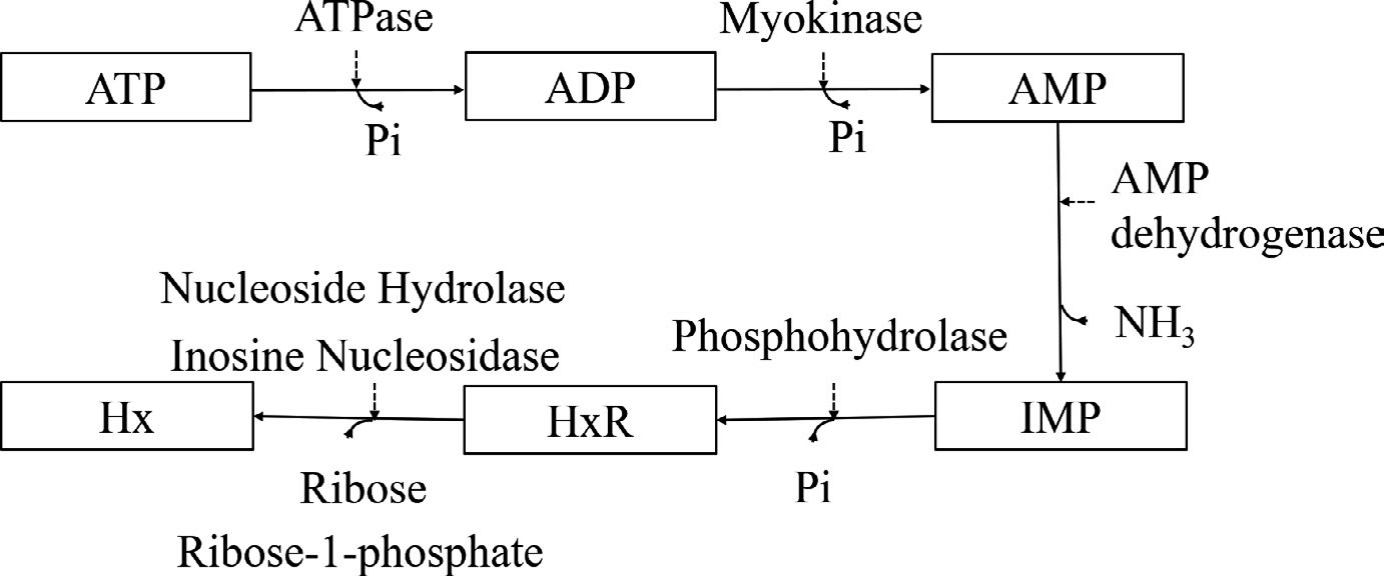
ATP‐related compounds metabolism in grass carp muscle

As shown in Figure 6, ATP is sequentially degraded into ADP, AMP, IMP, HxR, and Hx. Table 1 showed the contents of various nucleotides and the total contents of the processed fish fillets with different microwave drying time.

**TABLE 1:** Effects of microwave drying time on the contents of nucleotide compounds in dried fillets

MW time (min)	Contents(mg/100 g)						
	IMP	ATP	ADP	HxR	Hx	AMP	Sum
0	2.94 ± 0.09^e^	29.84 ± 1.31^a^	8.60 ± 1.33^c^	0.93 ± 0.08^d^	207.51 ± 15.89^a^	2.56 ± 0.17^a^	252.38 ± 18.20^c^
2	25.01 ± 1.38^d^	24.46 ± 3.08^bc^	8.42 ± 1.05^c^	1.12 ± 0.22^d^	171.04 ± 19.60^b^	2.43 ± 0.22^a^	232.48 ± 25.35^c^
4	214.69 ± 12.19^c^	21.63 ± 1.10^c^	21.14 ± 0.89^b^	4.72 ± 0.40^c^	129.61 ± 8.94^c^	1.53 ± 0.11^b^	393.32 ± 23.50^b^
6	378.93 ± 8.24^a^	25.86 ± 1.06^b^	28.06 ± 1.17^a^	7.24 ± 0.10^a^	74.57 ± 1.83^d^	1.01 ± 0.02^c^	515.67 ± 12.37^a^
8	294.73 ± 2.80^b^	23.88 ± 0.53^bc^	22.33 ± 1.30^b^	5.40 ± 0.15^b^	60.50 ± 1.06^d^	0.80 ± 0.01^cd^	407.64 ± 5.42^b^
10	280.51 ± 6.28^b^	30.32 ± 0.90^a^	24.21 ± 0.27^a^	4.98 ± 0.30^bc^	58.68 ± 0.90^d^	0.72 ± 0.01^d^	399.42 ± 8.54^b^

Note

different lowercase letters “a,” “b,” “c,” “d,” and “e” in the same row show that the index was significantly different (*p* < .05) at different time points.

The total nucleotide contents were significantly different (*p* < .05) in fillets dried with different microwave drying time, due to degradation reaction rates and molecular weights of nucleotides. In fresh grass carp (Wang et al., 2018), the conversion rate of IMP to HxR was very slow, resulting in high content of IMP and low of Hx. However, single hot‐air drying and short‐term microwave combined drying accelerated the decomposition of IMP and formation of Hx. Besides, the molecular weights of nucleotides were different (IMP: 348.21 g/mol; Hx: 136.11 g/mol). The 6‐min microwave‐drying fillets had the highest total nucleotide contents due to the large amount of IMP, while 0‐min and 2‐min fillets had the lowest due to Hx.

IMP imparts umami taste to fish and has synergistic effects with sweet amino acids in food to increase umami taste. The IMP content in fresh grass carp muscles was extremely high (Yang et al., 2019), but varied significantly (*p* < .05) in dried grass carp fillets at different microwave drying time. The IMP content in 0‐min microwave‐drying fillets was the lowest, only accounting for 1.16% of the total content, but the highest in 6‐min fillets (73.48%). Compared with fish fillets prepared by single hot‐air drying (0 min), the IMP content in 6 min group was increased by 129 times. Hx is the end product in ATP degradation, and its large accumulation would cause bitter and other unpleasant taste. The amount of Hx is largely determined by the IMP degradation rate. The Hx content was negatively correlated with the microwave drying time and reached the highest in the single hot‐air drying fillets (0 min). In addition, there was no significant difference (*p *＞ .05) in the 6 ~ 10 min microwave‐drying fillets, and the Hx content was much lower than 0 ~ 4 min microwave‐drying fillets. Combined with Figure 6, microwave treatment could inhibit biological enzyme activity (Al‐Harahsheh et al., 2009), which might explain the negative correlation between the Hx content and microwave drying time.

**FIGURE 7:**
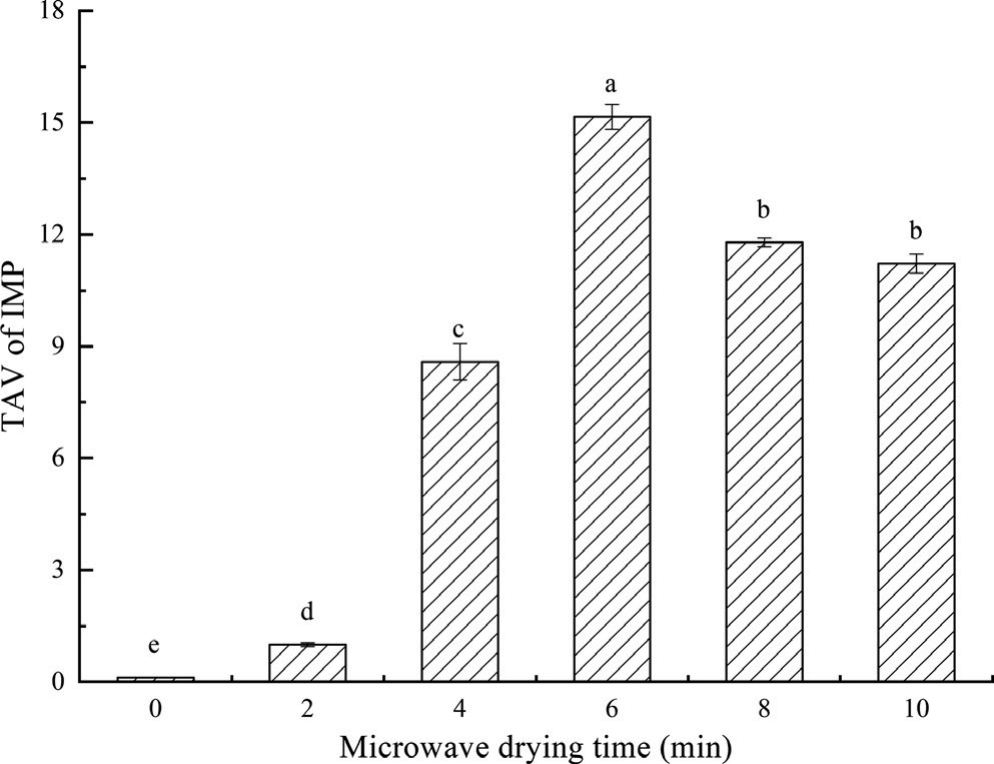
Effects of microwave drying time on TAV of IMP of dried fillets. The same index letters indicate that the mean values are not significantly different at a confidence level of 95% (*p* < .05)

Figure 7 showed the effects of different microwave drying time on TAV of IMP in combined dried fillets. The threshold of IMP is 25 mg/100 g (Ngapo & Vachon, 2016). TAVs were ranked as 6 >8 min ≈ 10 >4 >2 >0 min. TAV in the 0‐min microwave‐drying fillets was the lowest and far smaller than 1, indicating that IMP in the fillets prepared by single hot‐air drying could not independently contribute umami taste, while in the other fillets were all greater than 1. In particular, TAV in 6‐min microwave‐drying fillets reached a maximum value of 15.16 ± 0.33 g/100 g. Hence, 6‐min microwave‐drying fillets contained the most total nucleotides, the most umami nucleotides, and the least bitter nucleotides.

### 3.6 Free amino acids (FAAs)

FAAs are easily absorbed for their small molecules and considered as important odorants in aquatic products (Chen & Zhang, 2007). In this study, there were 17 FAAs detected in fish fillets. The contents, thresholds, and taste characteristics of FAAs were shown in Table 2.

**TABLE 2:** Effects of microwave drying time on the contents of FAAs in dried fillets

Amino acid species	Taste chara cteristics	Threshold (mg/100 g)	Contents (mg/100 g)					
			0 min	2 min	4 min	6 min	8 min	10 min
Asp	umami (+)	3	5.57 ± 0.08^a^	4.77 ± 0.05^b^	2.36 ± 0.13^c^	2.20 ± 0.19^c^	1.57 ± 0.16^d^	1.33 ± 0.14^d^
Thr	sweet (+)	260	40.62 ± 0.09^a^	33.21 ± 0.40^b^	39.65 ± 2.41^a^	42.09 ± 4.31^a^	26.88 ± 2.55^c^	18.50 ± 1.06^d^
Ser	sweet (+)	150	15.57 ± 0.00^a^	12.92 ± 0.06^b^	12.77 ± 0.72^b^	14.21 ± 1.46^ab^	8.81 ± 0.89^c^	6.61 ± 0.32^d^
Glu	umami (+)	5	16.21 ± 0.07^a^	14.06 ± 0.20^b^	8.69 ± 0.62^c^	7.34 ± 0.63^d^	5.35 ± 0.59^e^	5.21 ± 0.38^e^
Gly	sweet/umami (+)	130	45.45 ± 0.04^a^	39.35 ± 0.34^c^	41.79 ± 1.51^b^	36.86 ± 0.18^d^	26.04 ± 1.03^e^	23.70 ± 1.60^f^
Ala	sweet/ umami (+)	60	53.35 ± 0.19^a^	49.92 ± 0.03^a^	46.89 ± 2.91^a^	51.62 ± 4.05^a^	38.19 ± 3.95^b^	27.71 ± 1.55^c^
Cys	bitter/sweet/surfur (−)	ND	1.62 ± 0.02^c^	1.42 ± 0.07^c^	1.11 ± 0.01^d^	2.94 ± 0.20^a^	2.06 ± 0.19^b^	0.61 ± 0.04^e^
Val*	sweet/bitter (−)	40	41.50 ± 0.35^a^	30.81 ± 0.69^b^	37.71 ± 2.73^a^	39.64 ± 4.31^a^	23.80 ± 1.90^c^	17.04 ± 0.84^d^
Met	bitter/sweet/surfur (−)	30	11.97 ± 0.20^a^	10.78 ± 0.07^b^	8.85 ± 0.45^c^	6.97 ± 0.78^d^	4.15 ± 0.37^e^	3.79 ± 0.17^f^
Ile*	bitter (‐)	90	18.44 ± 0.26^a^	17.28 ± 0.10^ab^	15.23 ± 1.05^b^	18.97 ± 2.60^a^	11.04 ± 0.97^c^	6.88 ± 0.34^d^
Leu*	bitter (−)	190	28.46 ± 0.37^a^	25.67 ± 0.18^a^	24.58 ± 1.61^a^	25.70 ± 3.08^a^	15.11 ± 1.29^b^	11.34 ± 0.42^c^
Tyr	bitter (−)	ND	32.06 ± 0.26^a^	26.84 ± 0.55^b^	24.14 ± 1.61^b^	24.09 ± 3.50^b^	12.60 ± 1.02^c^	11.79 ± 0.52^c^
Phe	bitter (−)	90	11.18 ± 0.94^b^	24.60 ± 0.22^a^	24.28 ± 0.60^a^	16.77 ± 5.60^b^	10.80 ± 0.83^b^	14.26 ± 0.24^b^
Lys	sweet/bitter (−)	50	57.60 ± 0.17^b^	47.31 ± 0.70^cd^	55.65 ± 2.81^bc^	67.72 ± 7.82^a^	42.16 ± 3.67^d^	28.74 ± 1.09^e^
His	bitter (‐)	20	313.61 ± 0.58^b^	278.39 ± 1.06^b^	308.61 ± 17.51^b^	409.54 ± 31.77^a^	305.99 ± 24.22^b^	186.26 ± 9.26^c^
Arg	sweet/bitter (−)	50	26.16 ± 0.33^a^	21.32 ± 0.01^b^	22.12 ± 1.68^b^	23.39 ± 2.42^ab^	14.53 ± 1.27^c^	10.50 ± 0.43^d^
Pro	sweet/bitter (+)	50	56.90 ± 0.30^a^	38.66 ± 1.44^b^	49.63 ± 1.95^a^	53.21 ± 6.51^a^	35.69 ± 3.27^b^	22.80 ± 0.00^c^

Note

(+) indicates pleasant taste; (‐) indicates unpleasant taste; ND indicates that the threshold was not detected; different lowercase letters “a,” “b,” “c,” and “d” in the same row show that the index was significantly different (*p* < .05) at different time points.

Asp and Glu are recognized as the main umami amino acids (UAAs) of aquatic products, and proved to have strong synergy with umami nucleotides (Ngapo & Vachon, 2016). Gly and Ala can present umami and sweet (Yang et al., 2019). In addition, the synergy between Gly and Glu could further increase the umami intensity of aquatic products (Weng & Sun, 2007). Pro and Thr, as sweet amino acids (SAAs), also help enhance the umami taste of dried fish (Zhang, Wang, & Liu, 2019). Ile, Leu, and His are typical bitter amino acids (BAAs), which can complicate the taste of aquatic products and add meaty characteristics to grass carp (Dang et al., 2015).

The thresholds of Thr, Ser, Gly, Ala, Met, Ile, Leu, Phe, and Arg were further higher than their contents, so they made little contribution to the taste of grass carp. The contents of Glu and His were both greater than their thresholds in fillets with different microwave drying time. Glu could independently contribute its umami taste to the dried fillets. Its threshold is 5 mg/100 g. And its content decreased significantly (*p* < .05) with the increase of microwave drying time. The Glu content in the 0‐min microwave drying fillets was the highest and in 8‐min and 10‐min fillets were the lowest. Besides Glu, the contents of other eight FAAs (Asp, Gly, Ala, Met, Ile, Leu, Tyr, and Pro) were also negatively correlated with microwave drying time. The His contents were the highest in all groups. Its threshold was 20 mg/100 g. Different from the trend in Glu, the His content reached the highest in 6‐min microwave‐drying fillets, but the lowest in 10‐min fillets.

The Lys content varied significantly (*p* < .05) with the microwave time in the combined drying, which was the highest in the 6‐min fillets. In addition, the contents of Lys in 0 ~ 6 min fillets were all higher than its threshold. Lys could react with the intermediates of fat oxidation and form brown pigments, resulting in the reduction in content (Nakamura, Toyomizu, & Sasaki, 1976). Combined with Figure 4, the decrease of Lys in this study might be explained. Moreover, the Cys content was the lowest in the 17 FAAs detected in fish fillets, which was not consistent with fresh and steamed grass carp (Wang et al., 2018). Different from other FAAs, Cys had ‐SH, an active group, which was prone to lose electrons during dry processing. Cys lost its amino acid properties after oxidation, which was probably the main reason for its unusual low content.

**FIGURE 8:**
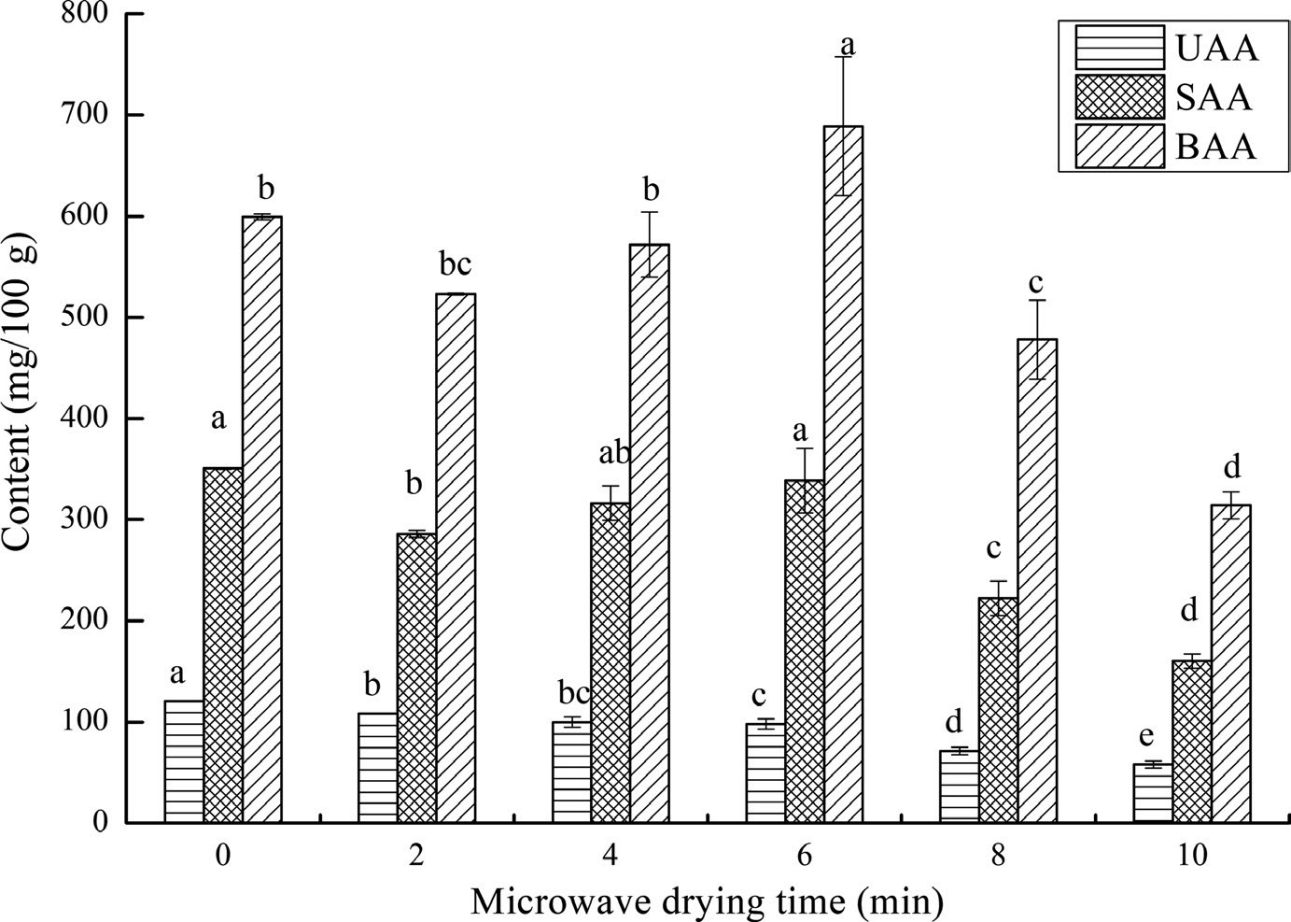
Effects of microwave drying time on the contents of different taste FAAs (UAAs, SAAs, and BAAs) in dried fillets. The same index letters indicate that the mean values are not significantly different at a confidence level of 95% (*p* < .05)

According to taste characteristics, FAAs were divided into UAAs, SAAs, and BAAs, of which the contents were shown in Figure 8. The BAAs content in dried fillets in each group was the highest, followed by SAAs, and UAAs was the lowest. The conclusion was consistent with FAAs in other dried fishes (Wang, 2018). Among BAAs, the His contents were much higher than other amino acids. It was noteworthy that the difference in His contents was considered to be one of the important reasons for the flavor change of fish (Yang et al., 2019). The SAAs contents were relatively high in the 0‐min and 6‐min microwave‐dried fillets. Unlike taste nucleotides, 6‐min microwave‐drying fillets had the highest BAAs content and 0‐min fillets had the highest UAAs content.

### 3.7 EUC and TAV

There is a synergistic effect between taste nucleotides and UAAs, which can further enhance the umami taste and improve the overall taste of aquatic products (Zhou, Wang, & Zhou, 2003). Besides sensory evaluation, EUC is the most widely accepted method for judging the umami intensity, and has been widely used to analyze aquatic products (Zhang et al., 2019).

**FIGURE 9:**
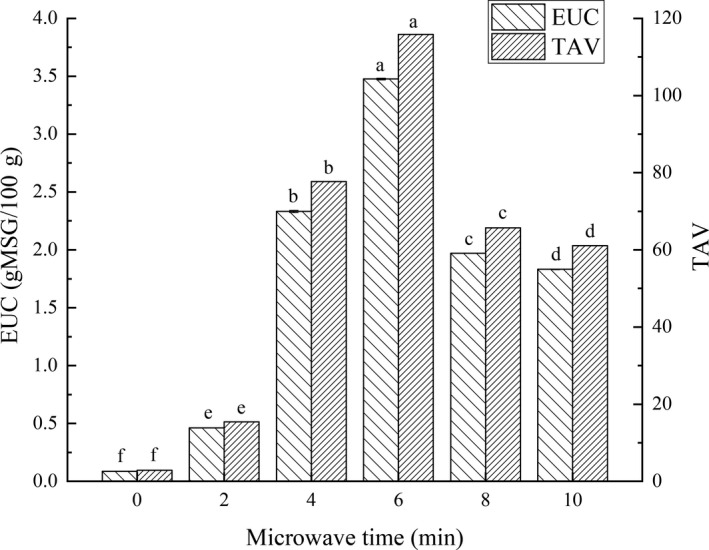
Effects of microwave drying time on the EUC values and TAVs in dried fillets. The same index letters indicate that the mean values are not significantly different at a confidence level of 95% (*p* < .05)

EUC and TAVs of grass carp fillets prepared by different microwave time combined with hot‐air drying were shown in Figure 9. The threshold of MSG was 0.03 g/100 g. Although the EUC was the lowest in 0‐min microwave‐dried fish fillets, the TAV was still greater than 1. Thus, the TAVs of EUC in other fillets were also greater than 1. It could be seen that the synergy between umami compounds greatly enhanced the umami intensity of dried fish fillets. Further, it was illustrated that the combined drying could significantly (*p* < .05) improve the synergistic effect of umami substances compared with the single hot‐air drying. The EUC of 6‐min microwave‐drying fillets reached the maximum, and its TAV was 40.6 times higher than that of the 0‐min fillets. Therefore, 6‐min microwave with hot‐air drying could greatly improve the umami taste of grass carp fillets.

The significant difference in IMP contents was the main reason for the EUC and TAV in fillets with different drying methods. The processing method had a great impact on the EUC of grass carp meat. It was proved that appropriate steaming processing could improve the EUC of grass carp belly and red meat (Yang et al., 2019). After the oil explosion treatment, the EUC of grass carp meat had also been improved (Jiang et al., 2015, 2019). In addition, different drying methods also made huge impacts on EUC. The EUC of *Pleurotus eryngii* treated with microwave drying was significantly (*p* < .05) reduced compared with hot‐air drying, probably due to overheating (Li et al., 2015).

### 3.8 Electronic tongue (E‐tongue)

**FIGURE 10:**
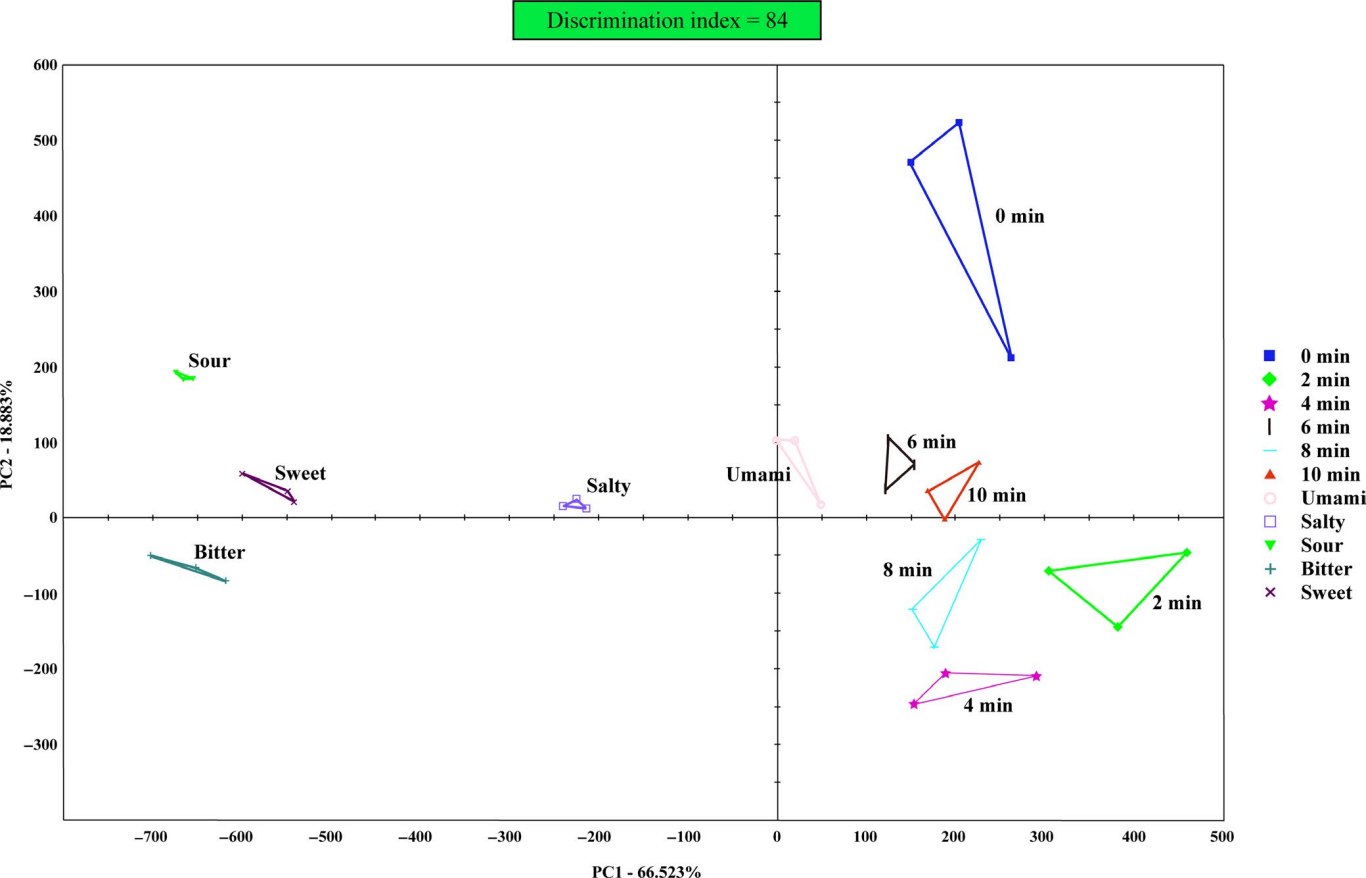
The PCA plot of E‐tongue response data on dried fillets with different microwave drying time (0, 2, 4, 6, 8, and 10 min) and five taste solutions (Umami, Salty, Sour, Bitter, and Sweet)

There was a significant correlation between sensory score determined by the E‐tongue and human sensory evaluation, which demonstrated that the E‐tongue could reliably characterize the taste (Fang et al., 2017). In this study, six groups of grass carp fillets (prepared by different microwave time combined with hot‐air drying) and five taste solutions (respectively representing umami, salty, sweet, bitter, and sour) were tested by the E‐tongue and displayed in the two‐dimensional scatter plot (Figure 10). The principal component analysis (PCA) method is used to extract multi‐index information with minimum variance information from a sensor network. If the cumulative variance contribution rate of PCA is greater than 85%, it can fully reflect the overall information of the sample (Garcia‐Hernandez, Salvo‐Comino, Martin‐Pedrosa, Garcia‐Cabezon, & Rodriguez‐Mendez, 2020). From Figure 10, the contribution ratios of PC1 and PC2 were respectively 66.52% and 18.88%, of which the sum was 85.40%. Hence, the PCA figure based on E‐tongue could fully reflect the taste information of dried fillets and taste solutions. The discrimination index (DI) in PCA figure is the ratio of the surface area of and between each group. If DI is positive, it means that the taste difference among each group is obvious. Otherwise, it is not. The maximum value of DI is 100. The closer it is to 100, the better the taste discrimination among each group. In this study, the DI was 84, indicating that the taste profiles of each group could be distinguished very clearly.

In Figure 10, the distance among groups could indicate the similarity in their taste profiles. The closer the two groups, the more similar the taste profile of the two groups (Legin, Rudnitskaya, Vlasov, Di Natale, & D'Amico, 1999). Among the five taste solutions, the umami solution was the closest to each dried grass carp fillets group, which meant umami prominent in fillets. The taste profile of 6‐min microwave‐drying fillets was the closest to the umami profile, but that of 0‐min fish fillets was the farthest. It indicated that the fillets with 6‐min microwave drying had the greatest umami intensity, while 0‐min fillets had the weakest. This result was consistent with the EUC in Figure 9. According to the results of EUC and E‐tongue, microwave‐hot‐air combined drying could make dried fillets show greater umami intensity than single hot‐air drying. Further, 6‐min microwave combined with hot‐air drying could produce the most umami grass carp fillets.

## 4 CONCLUSION

The study indicated that microwave combined with hot‐air drying was superior to single hot‐air drying on the quality of dried grass carp fillets. Furthermore, with the microwave power (385 W) and the hot‐air temperature (65°C) unchanged, 6‐min microwave drying time was the optimal microwave treated time for the fillets in consideration of all aspects. The 6‐min microwave‐drying fillets had the least total drying time (636 min), the lowest hardness (1,273.8 g), the brightest, and the whitest color (L*, 49.9; W, 48.3), and the highest rehydration rate (1.61). The combination of ATP‐related compounds, FAAs, EUC, and E‐tongue taste profiles, its umami taste was the most prominent among all groups for a large number of IMPs and corresponding synergistic effects. Despite the high content of BAAs, it had the lowest Hx content. In addition, it also retained quite a lot of SAAs, which could greatly improve the overall taste of fillets. In theory, the study enriched the drying process methods of low‐value aquatic products. From a practical perspective, the study laid the foundation for the production and application of microwave combined with hot air segmented drying for grass carp fillets in the near future.

## CONFLICT OF INTEREST

We declare that we do not have any commercial or associative interest that represents a conflict of interest in connection with the work submitted.
